# A Rapid High Throughput Vibration and Vortex-Assisted Matrix Solid Phase Dispersion for Simultaneous Extraction of Four Isoflavones for Quality Evaluation of *Semen Sojae Praeparatum*

**DOI:** 10.3389/fphar.2020.590587

**Published:** 2020-10-30

**Authors:** Xuejing Yang, Ali Sun, Evans Owusu Boadi, Jin Li, Jun He, Xiu-mei Gao, Yan-xu Chang

**Affiliations:** ^1^ State Key Laboratory of Component-based Chinese Medicine, Tianjin University of Traditional Chinese Medicine, Tianjin, China; ^2^ School of Pharmacy, Harbin University of Commerce, Harbin, China; ^3^ Tianjin Key Laboratory of Phytochemistry and Pharmaceutical Analysis, Tianjin University of Traditional Chinese Medicine, Tianjin, China

**Keywords:** isoflavones, high throughput ball mill, vibration and vortex-assisted MSPD, *semen sojae praeparatum*, high performance liquid chromatography

## Abstract

Isoflavones (daidzein, daidzin, genistein and genistin) were main chemical components and usually selected as markers for quality control of Traditional Chinese Medicine *semen sojae praeparatum* (SSP). High throughput vibration and vortex-assisted matrix solid phase dispersion and high performance liquid chromatography with diode array detection were developed to simultaneously extract and quantify four isoflavones in SSP. Some parameters influencing extraction efficiency of isoflavones by vortex-assisted matrix solid phase dispersion such as sorbent type, ratio of sample to sorbent, crushing time, vibration frequency, methanol concentration, eluting solvent volume and vortex time were optimized. It was found that the best extraction yields of four isoflavones were obtained when the sample (20 mg) and SBA-3 (40 mg) was crushed by ball mill machine for 2 min at vibration frequency of 800 times per minute. Methanol/water (1.5 ml, 8:2, *v/v*) solution was dropped into the treated sample and vortexed for 3 min. The recoveries of the four isoflavones ranged from 86.1 to 94.8% and all relative standard deviations were less than 5%. A good linearity (*r* > 0.9994) was achieved within the range 0.5–125 μg/ml. It was concluded that the high throughput vibration and vortex-assisted matrix solid-phase dispersion coupled with high performance liquid chromatography was user-friendly extraction and quantification method of multiple isoflavones for quality evaluation of SSP.

## Introduction

Traditional Chinese medicines (TCMs) have been used to treat the diseases for thousands of years. Biological activities of TCMs with therapeutic or toxic effect largely attributed to multiple effects of numerous components in TCMs (Yang et al., 2011). From this perspective, the great attention should be paid to simultaneous extraction and purification of target components from TCM samples for purposes of guaranteeing their efficacy and safety. Furthermore, the procedure for sample preparation is a high-priority step in analytical methods owing to the complexity of TCMs matrices and relatively low active components in TCMs. Conventional sample pretreatment techniques for extracting components in TCM include ultrasonic extraction, heat reflux extraction, soxhlet extraction, solid phase extraction, liquid-liquid extraction and microwave-assisted extraction (Yang et al., 2011; Xiong et al., 2009; Wu et al., 2012; Xiong et al., 2012; Yue et al., 2018; Akbari et al., 2019). However, these procedures are usually time consuming, inefficient, costly and require large volumes of organic solvents. In view of these shortcomings, it is invaluable to develop a simple, green and efficient pre-concentration method for components analysis of TCMs.

*Semen sojae praeparatum* (SSP) is a famous TCM for treating febrile fever, cold fever, headache, irritability and chest tightness in the Chinese pharmacopoeia (Chinese Pharmacopoeia Commission, 2020). It is made from fermented matured seed of soybean (*Glycine max* (L.) Merr). It was also regarded as an important food material of the traditional Chinese diet by Chinese communities around the world (Guo et al., 2018). According to the Classic books of TCM “Jin-Kui-Yao-Lue” and “ShangHanLun,” SSP was the main component of classical formula “Zhi-Zi-Da-Huang Tang,” “Zhi-Shi-Zhi-Zi-Chi-Tang,” and “Zhi Zi Chi Tang,” which have been clinically utilized for the treatment of alcoholic hepatitis, abdominal distension and depression for more than thousand years (Qu et al., 2014; Chen et al., 2015; Wang et al., 2016; Wang et al., 2019). It was reported that SSP could reduce oxygen consumption in the heart muscle, improve microcirculation, and treat tumors and osteoporosis (Qu et al., 2007; Chai et al., 2019). Genistein, genistin, daidzin, and daidzein were the main isoflavones of SSP due to its extensive biological activities. Isoflavones were known as natural phytoestrogens because their structure is similar to the structure of estradiol. They could simulate the bidirectional regulation of endocrine levels (Chai et al., 2017; Yao et al., 2018). Phytoestrogens, especially distributed in legumes, could replace the role of estrogen and prevent the occurrence of adverse reactions to estrogen. Experience in TCM shows that the herbal medicine containing isoflavones were often used to treat the depressurization, hypoglycemic, lipid-lowering, prevent disease of heart head blood-vessel and atherosclerosis, etc. Phytoestrogens in prevention and treatment of menopausal syndrome, osteoporosis after menopause, etc., are similar to the positive role of estrogen replacement therapy (Vitale et al., 2013; Cederroth and Nef, 2009). Therefore, it was meaningful to develop the efficient extraction and quantitative determination method of these bioactive isoflavones for improvement of quality evaluation of SSP (Mocan et al., 2018).

Matrix solid-phase dispersion (MSPD) was proposed and pioneered by Barker (1989), which was a robust technology for simultaneous extraction and clean-up of target components from solid or semi-solid matrices in a single step (Barker et al., 1989). It has been widely applied to detection of various target components in plant matrices, animal tissues, food stuff, sludge, aquatic biota, soil and indoor dust samples (Dorota and Marta, 2019). MSPD is a modified solid-phase extraction (SPE) method that requires manual blending of sample with solid abrasive material before elution of target components with fitting solvent. This technique is too cumbersome to enhance efficiency as well as simplify the analytical procedures. Vortex-assisted MSPD (VA-MSPD), which is based on substitution of the SPE step for a vortex agitation procedure, has demonstrated satisfactory results (Caldas et al., 2013; Escarrone et al., 2014; Hertzog et al., 2015; Du et al., 2018). Subsequently, a vortex-homogenized MSPD (VH-MSPD) has been established to determine halogenated phenolic compounds in seafood (Chen et al., 2016a) and short chain chlorinated paraffin from indoor dust samples (Chen et al., 2016b). This modified MSPD used vortex agitation procedure as mixing and blending steps instead of mortar and pestle for blending and homogenization. Recently, a combination of these two simplified techniques termed as dual-vortex-assisted MSPD, has been used to determine nine parabens in door dust (Chung et al., 2019). In spite of these improvements which made operation more convenient, the aforementioned techniques require transfer procedure between extraction (blending) and clean-up (the SPE) steps, which may result in quantitative loss of analytes. Subsequently, new procedure, balls-in-tube MSPD was proposed and used to determine 133 pesticide residues in apple, peach pear and plum. In this method, all sample preparations were done directly in closed extraction tube with the assistance of steel balls (Kemmerich et al., 2019). Preprocessing process of balls-in-tube MSPD required manual operation which is less efficient and time consuming especially when large batch of samples need be processed. Accordingly, the more robust, high-efficiency and controllable blend method is in great request.

Vibrating ball mill also known as tissuelyser or mixer mill is an equipment with two high-speed large-amplitude arms which grinds, mixes and breaks cells in seconds or minutes through grinding balls impacting and friction within the bowl. It is suitable for milling and homogenizing soft, fibrous, hard and brittle materials in the wet and dry state. Various kinds of samples ranging from plants, food, tissue of human and animals, hair have been crushed using vibrating ball mill (Sun et al., 2018). In other literatures, sea foods and human hair were crushed with ball mill but grinding was manually done with mortar and pestle (Moreda-Pineiro et al., 2012; Miguez-Framil et al., 2013). The extraction (blending) step was accomplished by substituting manual grinding with ball milling.

**FIGURE 1:**
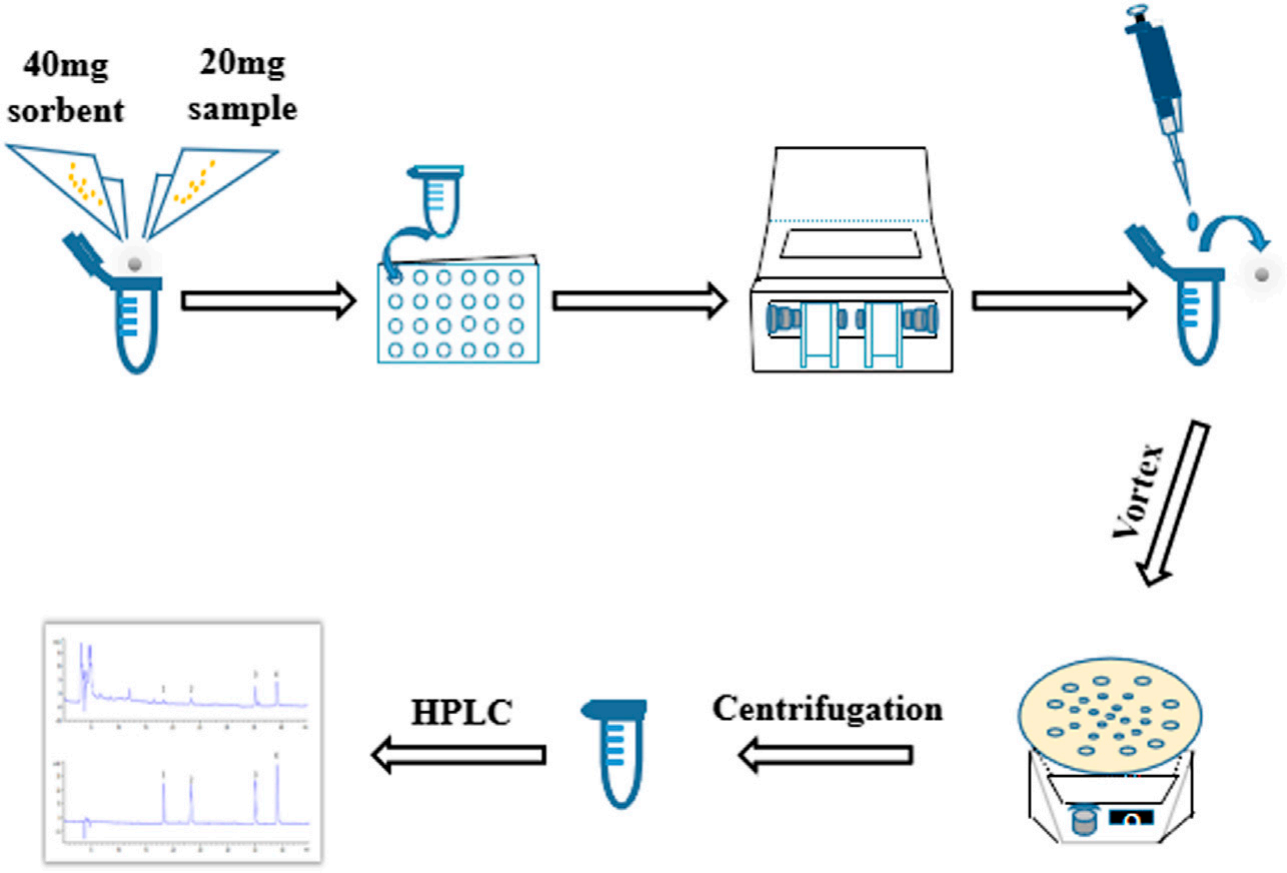
Schematic diagram of vibration and vortex-assisted MSPD method.

In present study, it aimed at optimizing a rapid, high throughput MSPD technique for detection of genistein, genistin, daidzein and daidzin in SSP. A modified MSPD method named as high throughput vibration and vortex-assisted matrix solid phase dispersion (VVA-MSPD) was proposed and established to extract and determine four isoflavones in SSP. All operations were performed in the same extraction tube in high throughput with ball mill machine vibration process. The vortex agitation was used to replace mixing, blending and SPE step, respectively. Besides minimization of manual grinding, the loss of analytes arising from transfer of mixed powder to SPE column or tube was reduced. Furthermore, ball mill has ability to handle dozens of simultaneous samples within seconds or a few minutes in which it might take hours by manual operation with great care and consistency. Additionally, main parameters affecting VVA-MSPD were systematically investigated to optimize this new method. In a nutshell, the newly established VVA-MSPD coupled with high performance liquid chromatography (HPLC) was successfully used to extract and detect the isoflavones for quality evaluation of SSP (Figure 1).

## Materials and Methods

### Chemicals and Reagents

HPLC grade methanol and formic acid were purchased from Fisher (Leicestershire, United Kingdom). Genistein, genistin, daidzein and daidzin (purity on HPLC > 98%) were supplied by Chengdu Desite Bio-Technology (Chengdu, China). C18 and Silica were provided by Welch Materials (Chatham Road Ellocott City, United States). SBA-3, TS-1 and MCM-41 were obtained from Nanjing JCNANO Technology (Nanjing, China). Ultrapure water was purified by a Grindi-Q Academic ultra-pure water system (Grindipore, Milford, MA, United States). All other reagents were of analytical grade.

### Herbal Plant

Five batches of SSP samples were obtained from the local pharmacy shops. All samples were identified as fermented processed product of matured seed of soybean [*Glycine max* (L.) Merr] by Prof. Yanxu Chang (Tianjin University Traditional Chinese Medicine). The material was ground through 65 mesh sieve, dried at 40°C and stored in a dryer.

### Apparatus and HPLC Analysis

Blending was done with a ball mill machine (TJG-25, Techin, China) with an adapter of 2 × 24 well plate that was fixed by fastening device, ceramic beads of 5 mm diameter and 2.0 ml micro-centrifuge tube (Beijing Labgic Technology Co., Ltd., Beijing, China).

Analysis was carried out on Agilent 1260 system (Agilent, Santa Clara, CA, USA) coupled with photodiode array detector (Scanning range 210–400 nm). Separation was performed on an Agilent Eclipse Plus C18 column (4.6 mm × 100 mm, 1.8 μm) connected with a LC-18 guard column (4.6 mm × 12.5 mm, 5 μm) at 30°C. Linear gradient elution system with acetonitrile (A) and ultrapure water (B) was as follows: 5–20% A at 0–10 min, 20–25% A at 10–25 min, 25–45% A at 25–32 min, 45–62% A at 32–37 min, 62–95% A at 40–45 min. Flow rate was set at 0.3 ml/min, injection volume of 2 μL for each run and detection wavelength of 258 nm.

### Preparation of Standard Solutions

Concentration of 1 mg/ml of each isoflavone (genistein, genistin, daidzein, and daidzin) in methanol solution (60%, v/v) was prepared. In addition, a mixed standard solution containing 0.25 mg/ml of each isoflavone was prepared and further diluted into range of concentrations for calibration curves.

### Vibration and Vortex-Assisted MSPD Procedure

Twenty milligram pulverized SSP and 40 mg sorbent (C18, silica, MCM-41, SBA-3 and TS-1) were mixed in a 2 ml round bottom centrifuge tube. A small ceramic ball was added and the lid covered tightly. The centrifuge tube was put into the adapter and installed on the ball mill machine to homogenize the mixture. Vibration frequency and crushing time were set at 800 times per min and 2 min, respectively. Ceramic ball was removed from the tube after the power settled.

One and an half milliliter methanol/water (8:2, v/v) was gently dropped into the tube and vortexed for 3 min. Thereafter, the solution was centrifuged at 550 g for 10 min. The eluted solvent was then filtered with 0.22 μm nylon membrane before HPLC analysis.

### Vibration-Forced MSPD Procedure

Initial procedures were same as outlined in VVA-MSPD method (“*Vibration and Vortex-Assisted MSPD Procedure*” section). After removal of ceramic ball, the mixture was transferred into an SPE column with a sieve plate at the bottom. Another sieve plate was gently blocked on the top with a glass rod. Methanol/water (8:2, v/v) was dropped into the tube to elute the volume to 1.5 ml after connection with a vacuum pump. The elute solvent was centrifuged at 550 g for 10 min and filtered with 0.22 μm nylon membrane before HPLC analysis.

### Normal Vortex-Assisted MSPD Procedure

Instead of ball milling procedure, 20 mg pulverized SSP and 40 mg sorbent (SBA-3) were mixed in an agate mortar, ground for 2 min to attain a homogenous mixture and then transferred into a centrifuge tube. Subsequent procedures were same as outlined in VVA-MSPD method.

### Ultrasonic Extraction

According to a previous study (Niu et al., 2010), 0.500 g SSP was ultrasonically extracted (40 kHz, 96% power) with 20 ml 50% (v/v) aqueous ethyl alcohol for 50 min. Thereafter, the lost weight was replenished with aqueous ethyl alcohol (50%, v/v) until reaction cooled down to room temperature. The solution was then filtered with 0.22 μm pore membrane before HPLC analysis.

### Heating Reflux Extraction

Heating reflux extraction was also performed in accordance with a previous study (Chai et al., 2014). Briefly, 1.000 g SSP was mixed with 25 ml 75% (v/v) aqueous methanol solution in a conical flask. The mixture was weighed, heat refluxed for 30 min, cooled down to room temperature and reweighed. Lost weight was made with 75% methanol (v/v). The solution was then shaken and transferred to a 25 ml volumetric flask and then brought to volume with 75% (v/v) aqueous methanol. The solution was filtered through a 0.22 μm pore membrane prior to chromatographic analysis.

### Optimization of VVA-MSPD Parameters

In order to obtain higher precision, sensitivity and efficiency of extracting isoflavones from SSP, seven parameters which can influence VVA-MSPD procedure were optimized. Specifically, type of sorbent (C18, silica, MCM-41, SBA-3 and TS-1); ratio of sample to sorbent (1:0, 2:1, 1:1, 1:2, and 1:3); crushing time of ball mill (1, 2, 3, and 4 min); vibration frequency of ball mill (500, 800, and 1,000 times per min); concentration of methanol (50, 60, 70, 80, 90, and 100% v/v); volume of eluting solvent (0.5, 0.75, 1, 1.25, 1.5, and 1.75 ml); and vortex time (1, 2, 3, and 4 min) were investigated. Each optimized parameter was further analyzed. All analytical samples were also analyzed in triplicate.

## Results and Discussion

### Effect of Related Factors in MSPD

#### Types of Sorbent

**FIGURE 2:**
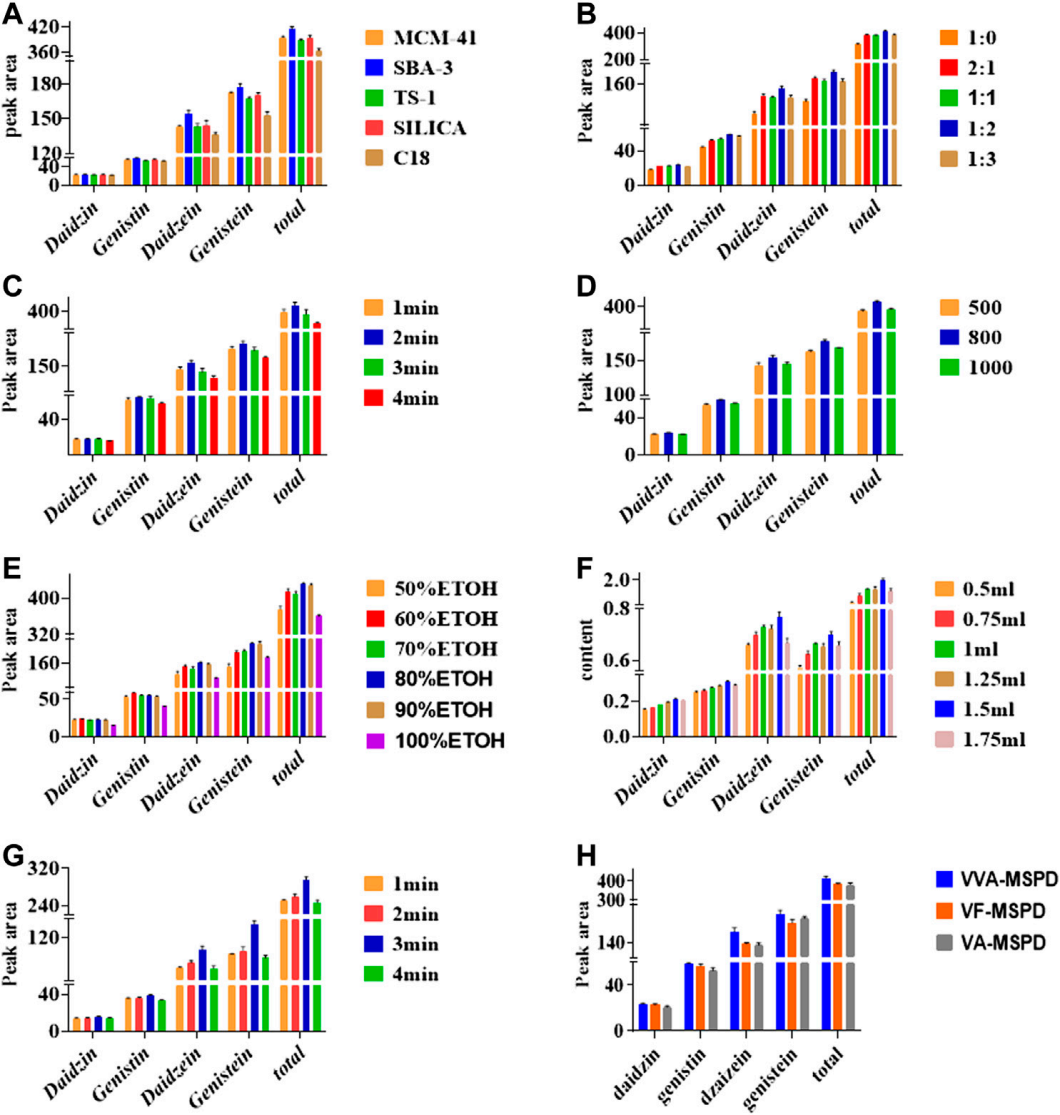
Effects of parameters on extraction of four compounds by single factor using VVA-MSPD technique: **(A)** type of sorbent, **(B)** mass ratio of sample to sorbent, **(C)** crushing time of ball mill, **(D)** vibration frequency of ball mill, **(E)** Selection of Methanol with different concentration, **(F)** volume of eluting solvent, **(G)** vortex time, **(H)** comparison of VVA-MSPD with VF-MSPD and VA-MSPD method. The errors bars represent RSD (*n* = 3).

Sorbent type is usually the main factor influencing sensitivity and selectivity of extraction the pre-analytical stage. Normal-phase material (silica), reversed-phase material (C18) and three molecular sieves (MCM-41, SBA-3 and TS-1) were selected to optimize the preferred sorbent. As shown in Figure 2A, the efficient of C18 was the least when the same ratios of liquid to solid remained constant. Comparatively, efficiencies of MCM-41, TS-1 and silica were similar whereas the efficient SBA-3 was the most in extracting isoflavones. SBA-3 was the preferred sorbent for further MSPD extraction procedure. SBA-3 has been applied in separation and preconcentration of crystal violet in water sample by a solid phase preconcentration procedure (Azarkohan et al., 2013). However, it has not been used in plants samples and MSPD process. This study indicated that SBA-3 was a suitable molecular sieve sorbent for MSPD procedure. Molecular sieves are crystalline metal aluminosilicates comprising of three-dimensional interconnecting networks of tetrahedral oxides.

#### Mass Ratio of Sample to Sorbent

Mass ratio is related to both interface area of sample matrix and dispersant as well as elution process. Results shown in Figure 2B indicated that peak areas of each target isoflavone increased gradually as mass of SBA-3 increased from 0 to 40 mg (1:0–1:2). With increased amount of SBA-3, more surface area and crystal cavities are available to target isoflavone. Interaction between sample matrix and sorbent gets higher, however, extraction yields of target isoflavone were slightly lower at ratio of sample to sorbent (1:3). Excess sorbent may lead to incomplete elution for the four isoflavones. In this regard, optimal sample to sorbent ratio of 1:2 was selected for further tests.

#### Crushing Time of Ball Mill

Crushing procedure supplied external forces to pulverize samples and facilitated interactions between samples and sorbents. Results presented in Figure 2C showed that the highest extraction efficiency was achieved for 2 min crushing time whereas prolonged crushing (from 3 to 4 min) gradually decreased yields of all isoflavones. Increment of crushing time may cause stronger interaction force between the target isoflavones and the dispersing sorbents, which make elution more difficult. Crushing time of 2 min was adapted for subsequent analysis.

#### Vibration Frequency of Ball Mill

Figure 2D showed that the extraction efficiency of target isoflavones was increased as vibration frequency was enhanced from 500 to 800 times per min. However, frequency of 1,000 times per min reduced extraction efficiency of the four isoflavones. Thus, the optimum vibration frequency was set at 800 times/min.

#### Selection of Methanol With Different Concentration

Selective and efficient desorption of isoflavones in complex mixtures could be achieved by selecting appropriate solvent polarity. It was found that extraction efficiency of isoflavones increased as methanol/water concentration increased from 50 to 80% (Figure 2E **)**. However, there was a slight decrease in yield with 90% methanol/water. Thus, methanol-water (80/20, v/v) was the best eluting solvent for VVA-MSPD procedure.

#### Volume of Eluting Solvent

Besides the choice of suitable solvent, moderate volume of eluting solvent during the desorption in VVA-MSPD procedure is also needed. The highest contents of all isoflavones were obtained when an elution volume was 500 μL (Figure 2F **)**.

#### Vortex Time

Transfer of the target components from solid phase to liquid phase could be accomplished by using optimum vortex. Figure 2G showed that each isoflavone had the highest peak at 3 min whereas extraction yields declined at 4 min vortex time. Finally, vortex time was set at 3 min.

### Method Validation

To verify the feasibility of the established VVA-MSPD coupled with HPLC-DAD method for determination of isoflavones in SSP*,* some parameters including limits of detection (LODs), limits of quantitation (LOQs), linearity, precision, repeatability, stability and recovery were estimated **(** Tables 1, 2 **)**. The established analytical technique was very sensitive with LODs (signal to noise ratio of 3:1) and LOQs (signal to noise ratio of 10:1) ranging from 0.25 to 0.40 μg/ml and 0.80–1.25 μg/ml, respectively. Good linearity (*r* > 0.9994) was achieved within the range 0.5–125 μg/ml. Intra-day precision at three level concentrations (2, 5, 50 μg/ml) ranged from 94.8 to 104% for accuracy and theirs RSDs were below 4.82% RSDs whereas inter-day precision results were 96.5–105% for accuracy and RSDs were below 4.32%. The sample stabilities varied from 96.2 to 104% and theirs RSDs were below 4.85%. The RSDs of repeatability was lower than 2.87%. The recoveries of target components ranged from 86.1 to 94.8% and theirs RSDs were below 5.00%. These results demonstrated that the proposed method could simultaneously quantify the four isoflavones in the sample of SSP.

**TABLE 1:** Linearity, LOD, LOQ, repeatability and recovery.

Component	Calibration curve	Test range (μg/ml)	*r*	LOD (μg/ml)	LOQ (μg/ml)	Repeatability (RSD%)	Recovery	
							Average (%)	RSD (%)
Daidzin	*y* = 8.5072*x*–7.7909	0.5–125	0.9994	0.13	0.5	2.75	86.1	4.74
Genistin	*y* = 12.248*x*–11.247	0.5–125	0.9995	0.12	0.5	2.87	90.6	4.63
Daidzein	*y* = 11.532*x*–8.4018	0.5–125	0.9997	0.25	0.8	1.83	94.8	5.00
Genistein	*y* = 15.683*x*–11.247	0.5–125	0.9995	0.07	0.2	1.35	93.7	4.24

**TABLE 2:** Accuracy and precison of intra-day and inter-day and stability for 24 h.

Component (μg/ml)	Concentration	Intra-day		Inter-day		Stability for 24 h	
		Accuracy (%)	RSD (%)	Accuracy (%)	RSD (%)	Remain (%)	RSD (%)
Daidzin	2	103	2.08	105	1.19	104	2.16
	5	104	4.25	105	2.31	104	3.41
	50	100	1.50	105	3.77	103	1.46
Genistin	2	102	1.66	104	1.06	103	1.73
	5	102	3.89	105	2.22	104	2.87
	50	97.0	1.47	100	1.39	98.7	1.36
Daidzein	2	94.8	3.38	100	1.83	97.30	1.96
	5	103	1.97	96.5	1.10	99.1	3.09
	50	96.2	0.64	96.1	1.61	96.2	1.56
Genistein	2	102	1.35	102	1.35	102	1.58
	5	102	4.82	102	4.32	102	4.85
	50	102	3.07	105	3.77	103	2.87

### Comparing VVA-MSPD to Other MSPD Methods

Figure 2H illustrates the extraction efficiencies of the developed VVA-MSPD, VF-MSPD and VA-MSPD methods. Individual peak areas as well as the total contents of all four isoflavones were the highest with our proposed VVA-MSPD method. Comparison with VVA -MSPD and VA-MSPD revealed that homogenization procedure of crushing with ball mill yields better extraction efficiency compared to hand grinding. Moreover, unlike VF-MSPD method, there was no quantitative loss of isoflavones with our developed method due to the absence of a transfer step. Comparatively, extraction efficiency of VVA-MSPD was higher than that of VF-MSPD.

### Sample Analysis and Comparison of VVA-MSPD With Traditional Extraction Methods

**FIGURE 3:**
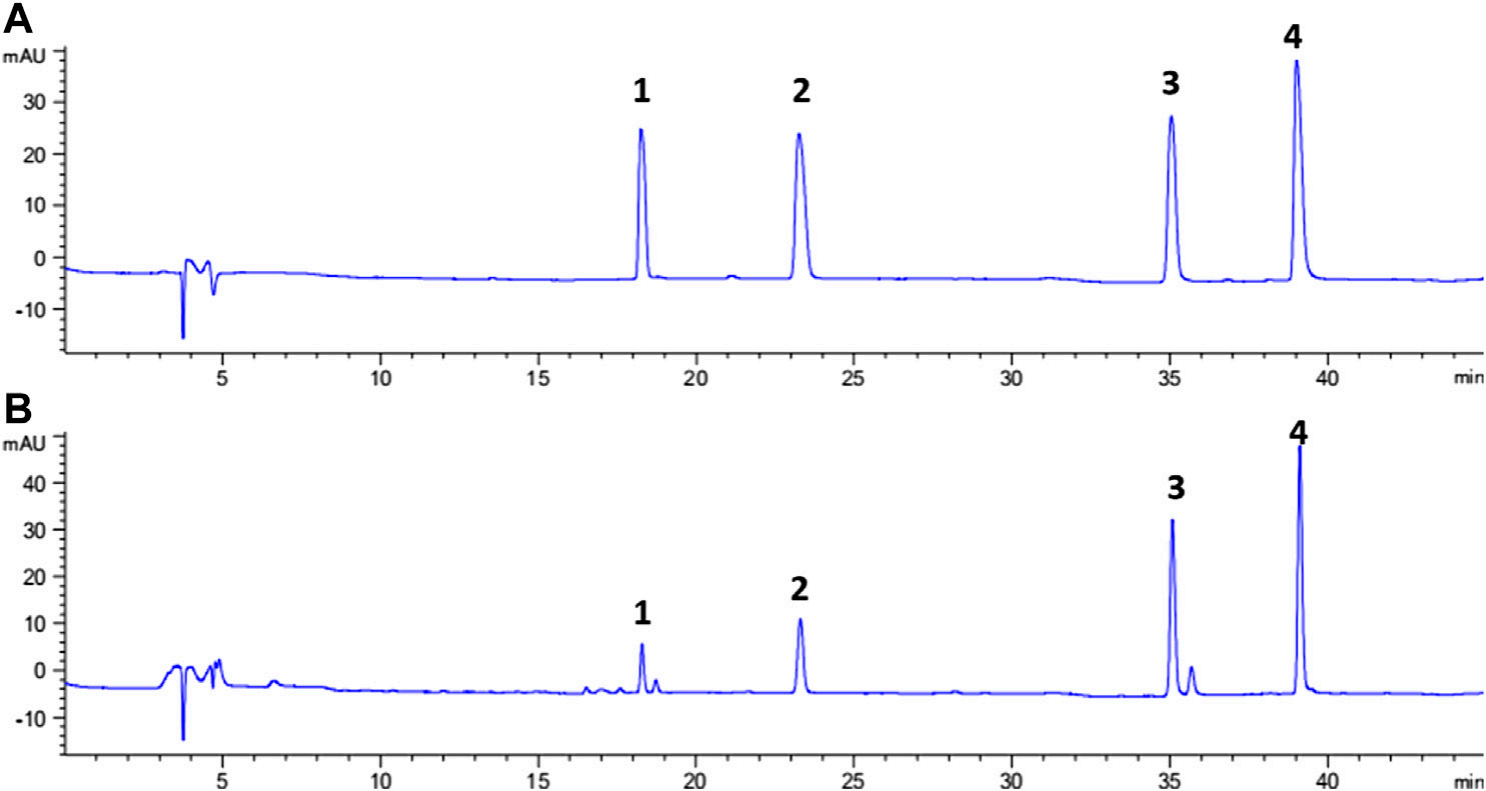
Typical chromatograms of standard solution of four analytes **(A)** and sample solution **(B)**, peak 1: daidzin; peak 2: genistin; peak 3: daidzein; and Peak 4: genistein.

Representative chromatograms showing the separation of the isoflavones and their contents in each batch of SSP are presented in Figure 3 and Table 3, respectively. Contents of four isoflavones in the five batches of different samples ranged from 0.21 to 1.56 mg/g for daidzin, 0.31–1.73 mg/g for genistin, 0.32–2.69 mg/g for daizein, and 0.48–2.41 mg/g for genistein, respectively. Furthermore, it was found that VVA-MSPD was higher than other conventional extraction methods such as ultrasonic assisted (Niu et al., 2010) and heating reflux (Chai et al., 2014) according to extraction efficiencies for all isoflavones in Samples 1, 1a, and 1b. Comparing to the corresponding yields by ultrasonic assisted extraction, extraction yields of daidzin, genistin daidzein and genistein were increased by 50%, 41%, 22% and 41% usingVVA-MSPD, respectively. The yields of daidzin, genistin daidzein and genistein by VVA MSPD extraction were 1.91, 1.82, 1.47 and 1.73 fold higher than those of daidzin, genistin daidzein and genistein by heating reflux method, respectively. It was concluded that the newly established VVA-MSPD method had outstanding extraction abilities for isoflavones in SSP. Genistein, genistin, daidzin, and daidzein are the main isoflavones of SSP. Among them, genistein is an effective anticancer substance, which could prevent, delay or block the occurrence of cancer through its multi-effect mechanism (Russo et al., 2016). Genistin has a variety of therapeutic effects, such as reducing the risk of osteoporosis and postmenopausal symptoms, anti-inflammatory, anticancer, heart protection, and antioxidant, etc (Liang et al., 2018; Islam et al., 2020). Daidzin could stimulate glucose uptake and has a hypoglycemic effect (Meezan et al., 2005). Daidzein has a protective effect on liver by inhibiting inflammation and oxidative stress and improving lipopolysaccharide induced hepatocyte injury (Yu et al., 2020). Therefore, it was of great significance to establish the efficient extraction and quantitative determination method of these bioactive isoflavones. The newly established VVA-MSPD method could provide an effective reference for the improvement of the quality standard of SSP.

**TABLE 3:** Contents of four components in semen sojae praeparatum samples by VVA-MSPD, Ultrasound and Heating reflux method (mg/g, *n* = 3).

samples	Daidzin	Genistin	Daidzein	Genistein
1	0.21 ± 0.01	0.31 ± 0.01	0.72 ± 0.01	0.76 ± 0.00
1^a^	0.14 ± 0.00	0.22 ± 0.00	0.59 ± 0.01	0.54 ± 0.01
1^b^	0.11 ± 0.00	0.17 ± 0.00	0.49 ± 0.01	0.44 ± 0.01
2	0.29 ± 0.00	0.46 ± 0.01	0.32 ± 0.00	0.47 ± 0.00
3	0.79 ± 0.01	1.30 ± 0.03	2.69 ± 0.05	2.41 ± 0.02
4	0.88 ± 0.02	1.17 ± 0.04	2.07 ± 0.10	1.08 ± 0.05
5	1.56 ± 0.04	1.73 ± 0.04	1.22 ± 0.05	0.82 ± 0.02

a Ultrasound extraction.

b Heating reflux extraction.

## Conclusion

A rapid, high throughput and effective VVA-MSPD based on SBA-3 method was successfully developed and extracted four isoflavones in SSP. Crushing sample by ball mill was introduced as a homogenization procedure whereas vortex agitation was used as SPE step in MSPD method. Combination of these two procedures ensured that all extraction processes occurred in the same tube. The quantitative loss of isoflavones was eliminated during transfer step as well as extraction efficiency was improved in the extraction procedure. It was demonstrated that VVA-MSPD coupled with high performance liquid chromatography is a rapid, high throughput and efficient method for extracting and determining the isoflavones and is a useful tool for quality evaluation of SSP.

## Data Availability Statement

All datasets presented in this study are included in the article/supplementary material.

## Author Contributions

YC, JL, and XG designed the experiment. YC and XY analyzed the experimental data. AS, XY, EB, JL, and JH performed the experiment. YC and XY wrote the manuscript.

## Funding

This research was supported by the National Key R&D Program of China (2019YFC1711000), National Natural Science Foundation of China (81973704 and 81374050), Science and Technology Program of Tianjin (No. 19ZYPTJC00060).

## Conflict of Interest

The authors declare that the research was conducted in the absence of any commercial or financial relationships that could be construed as a potential conflict of interest.
